# Evaluation of a Portable Automated Serum Chemistry Analyzer for Field Assessment of Harlequin Ducks, *Histrionicus histrionicus*

**DOI:** 10.4061/2010/418596

**Published:** 2010-02-22

**Authors:** Michael K. Stoskopf, Daniel M. Mulcahy, Daniel Esler

**Affiliations:** ^1^Environmental Medicine Consortium and Department of Clinical Sciences, College of Veterinary Medicine, North Carolina State University, 4700 Hillsborough Street, Raleigh, NC 27606, USA; ^2^U.S. Geological Survey, Alaska Science Center, 4210 University Drive, Anchorage, AK 99508, USA; ^3^Centre for Wildlife Ecology, Simon Fraser University, 5421 Robertson Road, RR1, Delta, BC, Canada V4K 3N2

## Abstract

A portable analytical chemistry analyzer was used to make field assessments of wild harlequin ducks (*Histrionicus histrionicus*) in association with telemetry studies of winter survival in Prince William Sound, Alaska. We compared serum chemistry results obtained on-site with results from a traditional laboratory. Particular attention was paid to serum glucose and potassium concentrations as potential indicators of high-risk surgical candidates based on evaluation of the field data. The median differential for glucose values (*N* = 82) between methods was 0.6 mmol/L (quartiles 0.3 and 0.9 mmol/L) with the median value higher when assayed on site. Analysis of potassium on site returned a median of 2.7 mmol/L (*N* = 88; quartiles 2.4 and 3.0 mmol/L). Serum potassium values were too low for quantitation by the traditional laboratory. Changes in several serum chemistry values following a three-day storm during the study support the value of on site evaluation of serum potassium to identify presurgical patients with increased anesthetic risk.

## 1. Introduction

Clinical chemistry parameters are routinely employed to evaluate the health of free-ranging wildlife including waterfowl, and baseline parameters have been published for many anseriformes in various situations [1–4]. Unfortunately, these parameters have been of limited use as cohort inclusion criteria, or for making other decisions in the field because of the difficulty of obtaining timely test results. Technological advances in serum chemistry analyzers have reduced the size and weight of equipment and simplified power source requirements, making field applications of real-time serum chemistry analysis more practical. We evaluated a portable analytical chemistry analyzer (VetScan, Sunnyvale, CA) capable of operating on 12 or 110 volt power and performing multiple blood tests from a single specimen for field assessment of serum chemistries of wild harlequin ducks (*Histrionicus histrionicus*) in comparison to a traditional laboratory analyzer. We also obtained some baseline serum chemistry values for the species and demonstrated the value of real-time serum chemistry analysis to identify ducks at increased anesthetic risk following a three-day storm.

## 2. Materials and Methods 

### 2.1. Experimental Animals

Harlequin ducks were captured for surgical implantation of intracoelomic radio transmitters with percutaneous antennae for telemetry studies of over-winter survival in Prince William Sound, Alaska. Capture was achieved using kayaks to herd groups of ducks rendered flightless by their annual wing molt into funnel traps placed near shore. A total of 198 blood samples (including duplicate samples from the same bird) were taken from 176 birds. The cohort for the establishment of baseline serum chemistry profiles was limited to 89 female ducks showing no overt clinical signs of disease, judged to be three years old or older by cloacal morphology [5], and whose serum samples had no visible hemolysis.

### 2.2. Sample Handling and Processing

A portable (6.9 kg, 29.2 cm long, 15.3 cm wide, 24.2 cm high) analytical chemistry analyzer (VetScan, Sunnyvale, CA) operated off 110 V service supplied by the boat's generator was used to obtain serum chemistry values onboard a chartered fishing boat. Prepackaged disposable reagent rotors included tests for potassium, glucose, alanine aminotransferase (ALT), albumin, alkaline phosphatase (ALP), amylase, calcium, cholesterol, creatinine, total bilirubin, total protein, and urea nitrogen. The device also evaluated and reported the amount of hemolysis and lipemia in the sample on a scale of 0 to 3+. Preliminary trials using mallard duck (*Anas platyrhynchos*) blood suggested the system developed for mammalian blood could be used with avian whole blood, plasma, or serum. 

Blood samples were drawn from the jugular vein of harlequin ducks immediately after collecting morphometric data including body weight in grams, and limb and bill measurements in millimeters from each newly captured duck. Fresh blood was analyzed when the burden of transmitter implantation surgeries allowed. Thirteen samples were suitable for comparison of whole blood with serum, meeting the criteria of no lipemia, icterus, or hemolysis greater than 1+ as determined by the VetScan equipment for the serum samples. All other samples were allowed to clot for 30 minutes prior to centrifugation at approximately 1000 G for 5 minutes for serum separation. Each serum sample was divided into two aliquots. Fresh serum was analyzed within 2 hours of sample collection using the VetScan system. The other aliquot was frozen at –10 C and transported to North Caroline State University. A matching panel of chemistries was performed for comparison on samples with no visible hemolysis using a Monarch 2000 (Instrumentation Laboratories, Lexington, MA). 

Samples with no visible hemolysis would occasionally result in a VetScan hemolysis rating of one plus. To evaluate the effect of sample hemolysis as rated by the VetScan, data from the 25 samples in the baseline cohort with hemolysis scores of 1+ were compared with data from the 64 samples with zero hemolysis reported by the VetScan and then against the combined cohort of 89 samples. Fifty two samples with hemolysis scores of 2+ and 15 samples with hemolysis scores of 3+ were compared separately with the 64 hemolysis score 0 samples in the baseline cohort.

Occasionally, individual chemistry determinations analyzed by one or the other system would fail or would report a concentration below detection limits. When this occurred, the data from each analyzer were excluded from comparisons for that sample parameter, resulting in different sample sizes for different parameters. 

The only time birds were detected with serum chemistry parameters sufficiently outside of expected ranges to warrant clinical concern was following a three-day storm characterized by high winds, which diminished sufficiently to permit capturing ducks late on the third day. Ten birds were captured on the day before the storm and 10 birds three days later as the storm abated. Another nine birds were caught the first full day following the end of the storm. Serum chemistry parameters for these 29 birds were extracted from the database and examined for differences potentially related to the storm. 

All data were summarized using the nonparametric descriptive statistics of medians and quartiles. The Wilcoxon Signed-Ranks Test and the Kolmogorov-Smirnov Two-Sample Test where used to compare data where appropriate. *P*-values less than  .05 were considered statistically significant.

## 3. Results

A total of 198 samples including duplicates and whole blood were processed with the VetScan under field conditions. Seventeen rotor failures occurred for an overall failure rate of 8.6%. The failure rate for whole blood samples was 14.3% compared to 7.6% for serum samples. Rotors failed twice on two samples but no samples were lost due to rotor failure, as a third analysis was successful in each case. There was no statistical correlation between degree of hemolysis or lipemia in the sample and rotor failure. 

VetScan hemolysis scores of 2+ or 3+ affected several serum chemistry parameters, as expected, but a reading of 1+ hemolysis had no statistically significant (*P* < .05) effect on the median value or variability of any of the serum chemistry parameters evaluated, including serum potassium. The use of no visible hemolysis as the inclusion criterion for the baseline determination study was appropriate. Hemolysis occurred less frequently in whole blood than in serum samples, and more frequently in the first days in the study, suggesting that handling the blood prior to analysis was the major contributor to hemolysis (data not shown). 

Baseline reference values for female harlequin ducks based on data from the VetScan and Monarch analyzers are presented in Table 1. Median values determined by each analyzer were within the quartile ranges of the respective alternative analyzer for glucose, calcium, and creatinine. Glucose baselines determined for the two analyzers were not different. The median values of most other parameters examined were lower when determined on the Monarch analyzer with the exception of calcium and creatinine, which were higher. The extents of the quartile ranges were similar for the two analyzers for most parameters. Serum potassium concentrations determined by the Monarch analyzer were frequently reported as the minimum level of quantitation resulting in a very narrow quartile range. Although relatively few duplicate samples were run, the precision of the VetScan data appeared to be similar to the precision of the data generated by the Monarch reference methods (data not shown). These results are compatible with those of studies comparing the VetScan with larger automated systems for assessment of serum chemistry values of parrots in clinical settings [6].

Thirteen samples with no hemolysis when analyzed as serum were also analyzed as whole blood on the VetScan equipment. Lipemia and icterus readings for all of these samples were zero; however 2 of the 13 whole blood samples registered 1+ hemolysis readings. The sign of the differences for total protein, total bilirubin, ALT, and creatinine concentrations analyzed from whole blood and serum were not consistent (Wilcoxon Ranked-Sign Test). Whole blood potassium concentrations were consistently lower than those from the same samples analyzed as serum (median difference: –1 mmol/L). Glucose, cholesterol, calcium, and amylase concentrations in whole blood were consistently higher than those determined from serum (Table 2). 

Serum chemistry parameters of 29 birds were examined around the storm event (Table 3). The most dramatic change, noted during field sampling, was the precipitously lower serum potassium concentrations in ducks captured immediately after the storm abated compared to ducks captured the day before the storm. Serum potassium levels in ducks captured the next day after the storm had abated on the third day of storm conditions had returned to within baseline quartiles. 

Median serum glucose concentration was also lower in ducks captured immediately after the storm abated. Although these data remained within the baseline quartiles established by the overall study, they were much less variable compared to samples taken on days without a storm. Serum glucose concentrations in ducks captured the next day after the storm abated were uniformly higher than for birds captured immediately before the storm, but again, they were within the baseline quartile values established by the survey study.

The only change other than serum potassium levels that resulted in values outside of the baseline quartiles for the parameter was an increase in median total protein in serum samples collected immediately following the storm which only partially returned to baseline in birds captured the next day after the storm abated. Other more subtle trends included higher median cholesterol in ducks caught immediately after the storm began to abate on day 3, which appeared to rebound, falling below expected baseline concentrations in ducks captured a day later. A similar pattern was seen for serum ALT (median after storm 43.5 U/L, data not included in Table 3). Median serum alkaline phosphatase was higher in ducks captured immediately after the storm abated (666.0 U/L), but then fell back to prestorm concentrations (500.0 U/L) in ducks captured a day later, but always remained within the baseline quartiles for the parameter. Similarly, median serum amylase concentrations in ducks captured immediately after the storm abated (1479.5 U/L) and a day later (1430.0 U/L) were higher compared to ducks captured immediately before the storm (1231.5 U/L) but remained within the baseline quartiles for the parameter based on the entire cohort of the survey study. 

## 4. Discussion

Analysis of blood samples taken from free-ranging wildlife is usually deferred until the samples can be sent to an analytical laboratory. Vagaries of initial processing, storage in the field, the unpredictable conditions and hazards of transportation, and storage and delays in the analytical laboratory can introduce unwanted variations in the data obtained. Also, results are typically not obtained until after the fieldwork is finished. The VetScan analyzer provided immediate results for a useful series of serum parameters. Samples were run as whole blood or serum, with a slightly higher, but acceptable, failure rate when whole blood was used. The small size of the equipment, its ability to use 12 V or 110 V power supplies, its automatic operation, and its durability to being moved and to field environmental conditions make real-time screening of surgical candidates possible. 

For consistency, we chose nonparametric descriptive statistics because the distributions of data for many of the parameters examined were distinctly non-Gaussian. The use of quartiles (25th and 75th percentiles) to establish baseline ranges departs from the more common practice with domestic animals to accept values between the 2.5th and 97.5th percentiles as within reference boundaries [7]. Using quartiles increases the likelihood of a Type I error of excluding truly “normal” animals from a study, but greatly reduces the chance of a Type II error where an abnormal is included in the “normal” cohort. This approach is useful when capture of animals is not the limiting factor in constructing the necessary cohorts for a study, or when significant effort is to be invested on an individual based on its presumed physical health or “normality.” 

Results of serum analyses using the VetScan machine were comparable to results obtained using a laboratory-based machine of a type typically found in large analytical laboratories. The results of our study emphasize the importance of establishing baseline ranges specific for the instrumentation being utilized. The VetScan analyzer had a greater sensitivity for analysis of samples with very low levels of potassium than did the Monarch analyzer. The VetScan also tended to return higher values for Alkaline Phosphatase, Alanine Transferase, and Amylase. The magnitude of these differences arguably would not affect clinical assessment of a patient, but awareness of the technology specific baseline ranges would reduce the chance of over interpretation. The Creatine values returned by the Monarch analyzer on aggregate were nearly double those returned by the VetScan, and this difference would be expected to impact clinical assessment. The comparison of values returned on the VetScan Analyzer from serum and whole blood from the same animal returned generally consistent results. The most marked differences were observed for the creatine assay, which obtained lower values consistently from serum samples. The differential in total protein determination we observed was sufficiently large to impact clinical assessment of a patient, but the variability in the direction of difference obscured any reliable data transformation for comparison of results between the different sample types. 

The impact of weather on birds feeding at sea is receiving considerable recent attention and thermodynamic/biophysical models suggest that high wind velocity can impact bird physiology [8]. In our study, serum glucose concentrations in ducks captured immediately after the storm abated on the third day were lower than those in birds captured before the storm, but the glucose concentrations of ducks captured a full day after the storm abated were higher than prestorm values. All of these changes remained within the quartiles determined for prestorm birds and for the entire baseline cohort, but there were interesting changes in variability. The data from post-storm birds were essentially uniform, with the quartiles of samples obtained the second day after the storm abated extending less than half the range of prestorm data. This is compatible with the hypothesis that ducks were unable to eat during the storm and were forced by the storm effects to concentrate on feeding immediately after the weather subsided. The increased serum total protein in birds caught after the storm was compatible with storm-induced hemoconcentration due to dehydration. A similar pattern was seen for serum ALT and alkaline phosphatase, which returned to baseline levels in ducks sampled two days after the storm had abated. Serum amylase and total serum calcium were also higher in birds caught immediately after the storm, but continued to increase in birds caught two days after the storm. Serum cholesterol increased, and then fell below prestorm levels. Although the initial effect could also be explained by hemoconcentration, the apparent strong, overcompensating rebound suggests endocrine modulation similar to what has been suggested in captured beluga whales [9]. 

Serum potassium concentrations were significantly lower in birds captured immediately after the storm abated than those of birds captured immediately prior to the storm. Individual birds in the immediate post-storm cohort had serum potassium concentrations below detectable limits of the VetScan analyzer (2.0 meq/L) and below concentrations routinely associated with adverse peri-operative events including serious arrhythmias and death in human surgical patients [10]. Potassium depletion can cause decreased intracellular fluid volume, changes in membrane potential, intracellular pH, and potassium dependent enzymatic reactions [11]. Such changes could affect survival during and following anesthesia and surgery [12–15]. Internal potassium balance is affected by acid-base status, exercise, and catecholamine release [16]. Catecholamine release routinely causes an initial mild hyperkalemia followed by hypokalemia because of beta adrenergic receptor responses [17–19]. A rapidly developing, profound hypokalemia can occur with reduced dietary intake [16]. Conservation of potassium by adjustments in renal ion management can be delayed for several days when animals that are normally on a high potassium diet are suddenly taken off feed or develop anorexia [20, 21]. We postulate that the serum chemistry differences in serum potassium, glucose and cholesterol observed after the three-day storm event can be readily explained by an inability of the birds to feed during the storm, and to an increase in physical exertion by the birds to cope with high winds and waves.

## 5. Conclusions

(1) On-site evaluation of serum chemistry parameters for pre-surgical screening of waterfowl in remote locations is feasible and offers the advantage of access of key data prior to making decisions on anesthesia and surgical risk for patients. (2) Serum potassium and glucose concentrations of Harlequin ducks can be impacted by environmental conditions in ways that may increase the risk of anesthesia and surgery. 

## Figures and Tables

**Table 1:** Female harlequin duck serum chemistry baseline reference values as determined on paired samples analyzed using the portable VetScan machine and a laboratory analyzer (Monarch).

		VetScan			Monarch		
Parameter	Units	*N*	Median	Quartiles	*N*	Median	Quartiles
Glucose	mmol/L	88	18.8	16.9 –20.4	82	18.4	17.0–20.2
Potassium	mmol/L	88	2.7	2.4–3.0	82	2.0	2.0–2.1
Alkaline phosphatase	U/L	86	500	291–770	82	253	142–377
Alanine transferase	U/L	88	35	26–47	82	17	14–22
Amylase	U/L	88	1430	1191–1635	82	978	827–1169
Total bilirubin	*μ*mol/L	79	3.4	3.4–5.1	1	3.4	—
Calcium	mmol/L	88	2.3	2.3–2.4	82	2.3	2.3–2.5
Cholesterol	mmol/L	88	5.6	4.5–6.5	82	5.3	4.3–6.2
Creatinine	*μ*mol/L	88	17.7	17.7–26.5	82	44.2	35.4–53.0
Total protein	g/L	88	39	36–41	82	34	32–37
Globulin	g/L	NA	NA	NA	82	21	19–23

**Table 2:** Comparison of paired samples of whole blood and serum using the VetScan analyzer (*N* = 13).

Parameter	Units	*R*	Median difference	Quartiles	Range of differences
Glucose	mmol/L	0.95	0.6	0.3–0.9	− 2.6 to 1.3
Potassium	mmol/L	0.70	−1.0	−0.6 – -0.6	−1.3 to -0.6
Alkaline phosphatase	U/L	0.99	6	0–17	−106 to 17
Alanine transferase	U/L	0.98	−1	−2–1	−3 to 2
Amylase	U/L	0.95	30	18–67	−225 to 289
Total bilirubin	*μ*mol/L	8.7	0.7	0–1.7	−1.7 to 1.7
Calcium	mmol/L	0.18	0.08	0.03–0.1	−0.28 to 0.18
Cholesterol	mmol/L	0.02	0.26	0.05–0.54	−0.67 to 0.98
Creatinine	*μ*mol/L	14.1	0	−8.8–8.8	−17.7 to 26.5
Total protein	g/L	4.6	2.0	1.0–3.0	−6.0 to 6.0

**Table 3:** Comparison of median serum calcium (mmol/L), potassium (mmol/L), and total protein (g/L), glucose (mmol/L), cholesterol (mmol/L), and alkaline phosphatase (IU/L) of female harlequin ducks from before (*N* = 10), immediately after (*N* = 10), and one day after (*N* = 9) exposure to a three-day storm (median winds exceeding 15 knots).

Parameter	Calcium	Potassium	Total	Glucose	Cholesterol	Alkaline
	(mmol/L)	(mmol/L)	Protein (g/L)	(mmol/L)	(mmol/L)	phosphatase (U/L)
Prestorm	2.3	2.6	37	18.3	5.6	499.5
Immediately after storm abated	2.4	2.0	42	17.7	6.4	666.0
24 hrs after storm abated	2.4	2.7	39	19.3	4.2	500.0
Baseline	2.3	2.7	39	18.6	5.6	506.5
